# Supplementary material for: The interactome of intact mitochondria by cross-linking mass spectrometry provides evidence for coexisting respiratory supercomplexes
Source: Mol Cell Proteomics. 2017 Dec 8;17(2):216–32. doi: 10.1074/mcp.RA117.000470 (PMC5795388; doi:10.1074/mcp.RA117.000470)
Supplement: Supplemental Data [file supp_RA117.000470_133922_0_supp_23978_fzffwf.zip › spectra_annotation/mito_spectra_annotation/498-1-2-1-18-1.pdf]

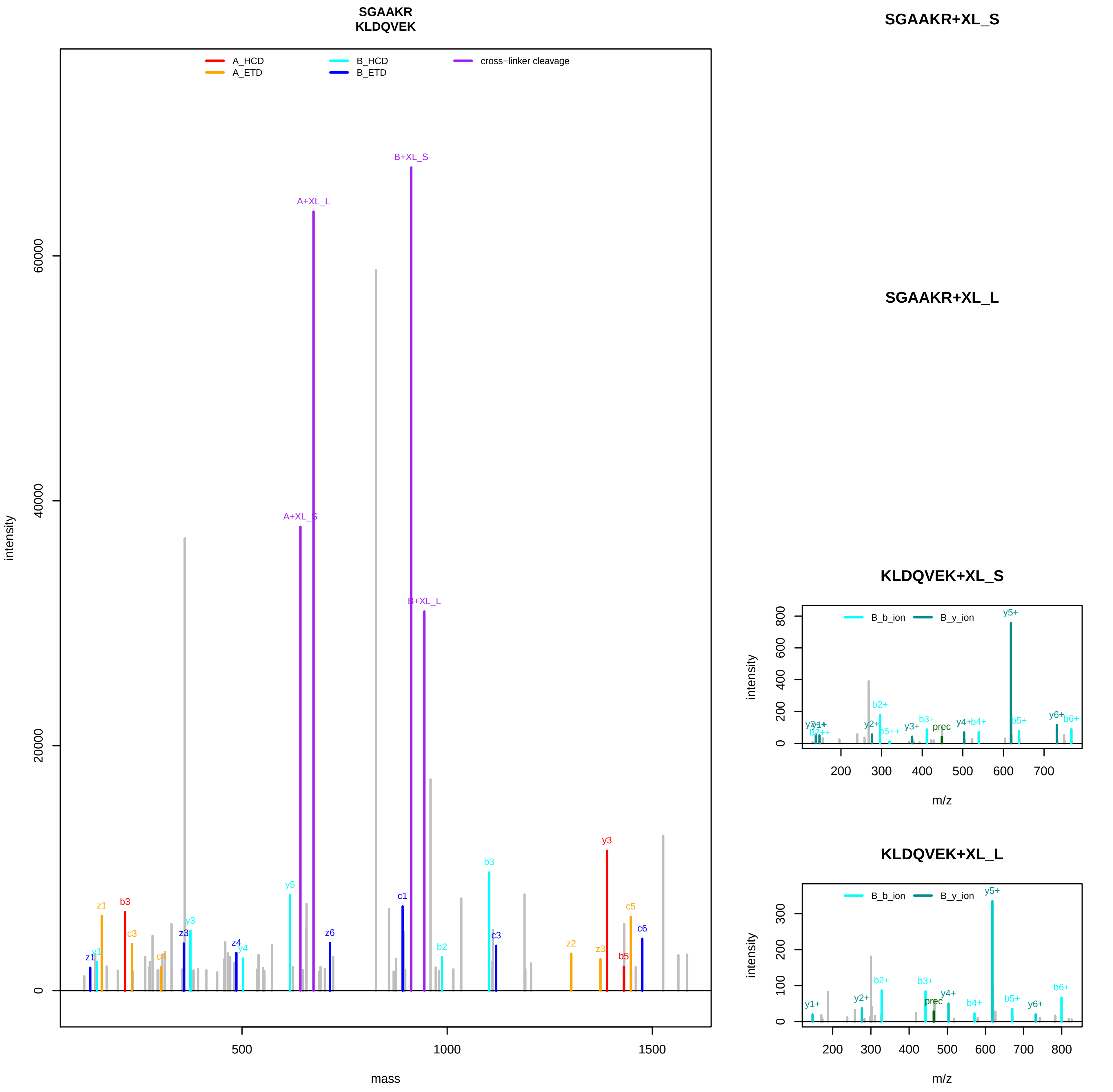
SGAAKR
KLDQVEK
A_HCD
A_ETD
B_HCD
B_ETD
cross−linker cleavage
B+XL_S
A+XL_L
A+XL_S
B+XL_L
z1
y1
z1
b3
c3
c4
z3
y3
z4
y4
y5
z6
c1
b2
b3
c3
z2
z3
y3
b5
c5
c6
intensity
mass
0
20000
40000
60000
500
1000
1500
SGAAKR+XL_S
SGAAKR+XL_L
KLDQVEK+XL_S
B_b_ion
B_y_ion
y5+
b2+
y2+
b5++
y3+
b3+
prec
y4+
b4+
b5+
y6+
b6+
intensity
m/z
0
200
400
600
800
200
300
400
500
600
700
KLDQVEK+XL_L
B_b_ion
B_y_ion
y5+
y1+
y2+
b2+
b3+
prec
y4+
b4+
b5+
y6+
b6+
intensity
m/z
0
100
200
300
200
300
400
500
600
700
800
